# Supplementary material for: Nucleotide proofreading functions by nematode RAD51 paralogs facilitate optimal RAD51 filament function
Source: Nat Commun. 2021 Sep 20;12:5545. doi: 10.1038/s41467-021-25830-x (PMC8452638; doi:10.1038/s41467-021-25830-x)
Supplement: Supplementary file 1 — Supplementary Information [file 41467_2021_25830_MOESM1_ESM.pdf]

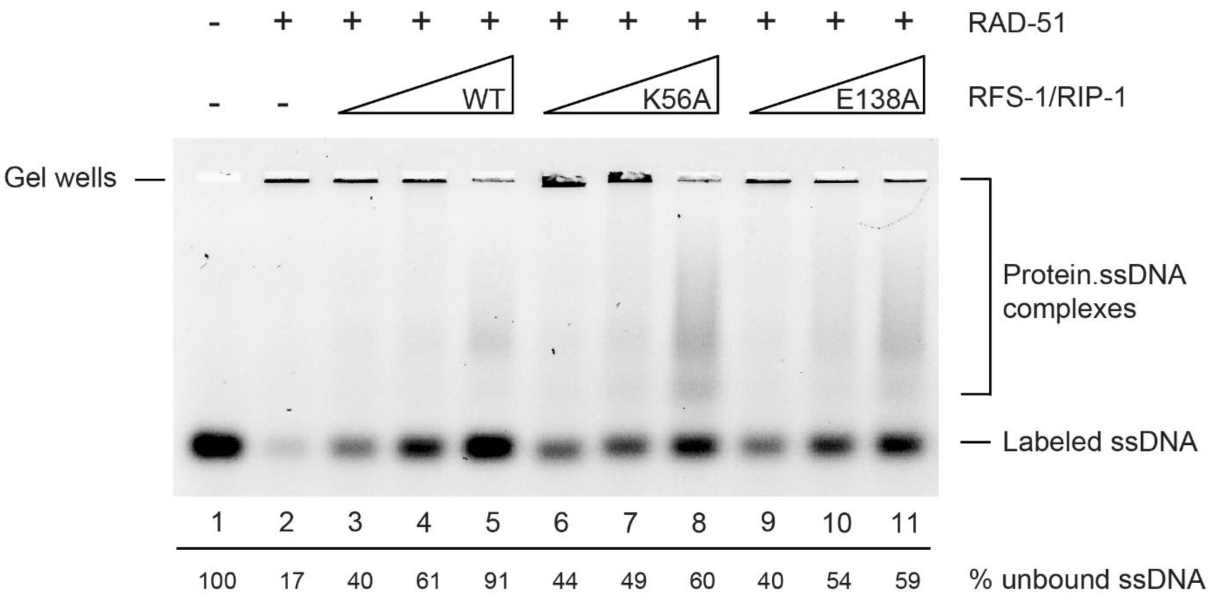

Supplementary Fig. S1 | Inhibition of RAD-51 binding to ssDNA by RFS-1/RIP-1 using EMSA. RAD-51 (1 µM) was pre-mixed with different concentrations (0.25, 0.5 and 1 µM) of RFS-1/RIP-1 (WT, K56A or E138A), and 5'-FITC-labelled 61mer ssDNA (10 nM) was added for 10 min. Protein-DNA complexes were crosslinked and resolved in an agarose gel. Representative gel is shown (n = 3). Source data are provided as a Source Data file.

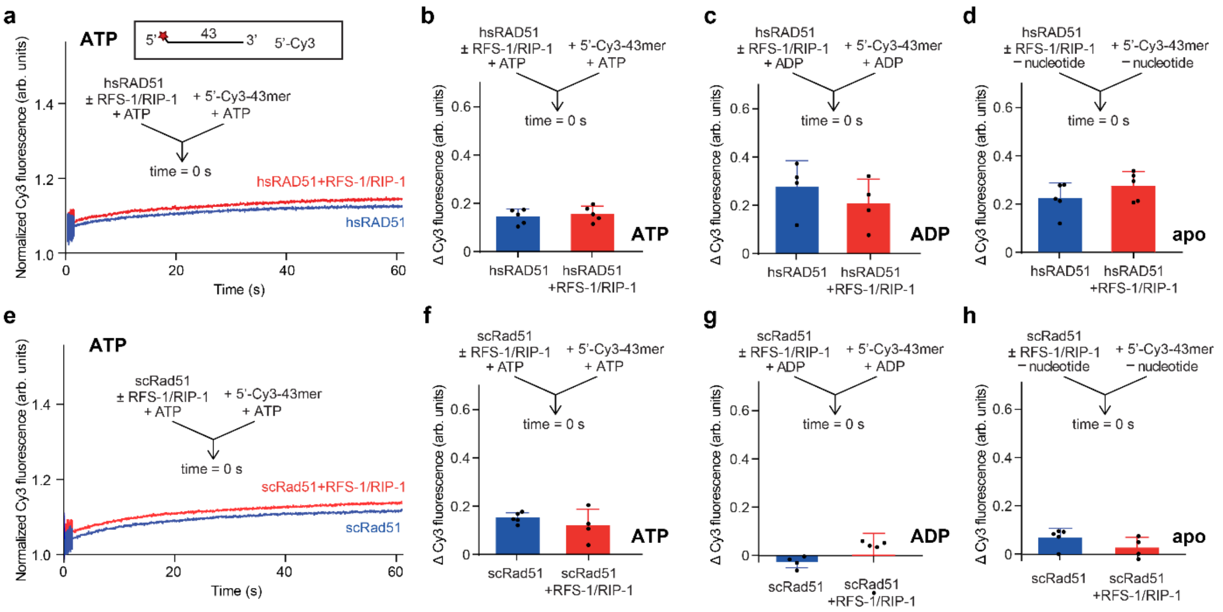

Supplementary Fig. S2 | Control of RAD-51 ssDNA binding by RFS-1/RIP-1 is species-specific. Effect of RFS-1/RIP-1 on formation of human RAD51 (hsRAD51, panels **a-d**) or yeast Rad51 (scRad51, panels **e-h**) ssDNA filaments in the presence of ATP (**b** and **f**) or ADP (**c** and **g**), or absence of any nucleotide (apo) (**d** and **h**). **a, e** Average normalized 5'-Cy3-43mer fluorescence profiles plotted as a function of time. The arrow indicates the components of the two syringes rapidly mixed at the 0 s time point in a stopped flow instrument. hsRAD51 or scRad51 alone (250 nM) or in the presence of RFS-1/RIP-1 (50 nM) was mixed with 5'-Cy3-43mer ssDNA (15 nM) in the presence of ATP and ADP, whereas 1000 nM for both proteins in the absence of nucleotide. **b-d, f-h** Bar graphs of average Δ Cy3 fluorescence for experiments performed under different nucleotide co-factor conditions (mean; errors: s.d.) (b: n=5, c: n=4, d: n=5, f: n=4-5, g: n=4-5, h: n=4-5). Source data are provided as a Source Data file.

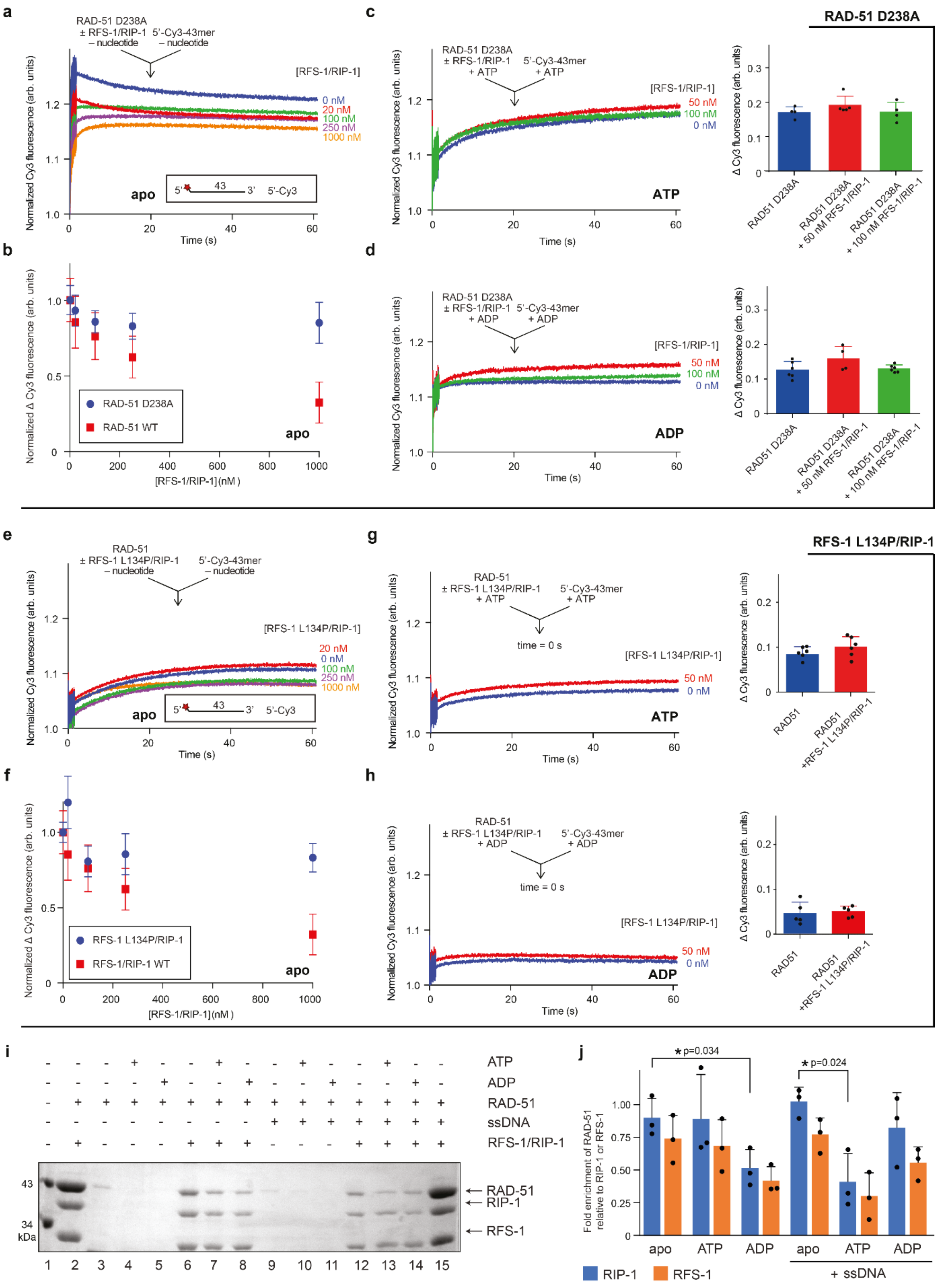

Supplementary Fig. S3 | Functional analysis of RAD-51 D238A and RFS-1 L134P/RIP-1 mutant proteins. a-d Effect of RFS-1/RIP-1 on RAD-51 D238A mutant ssDNA binding in the absence of nucleotide (**a-b**) or in the presence of ATP (**c**) or ADP (**d**). **a**, **c**, **d** Average normalized 5'-Cy3-43mer fluorescence profiles plotted as a function of time. The arrow

indicates the components of the two syringes rapidly mixed at the 0 s time point in a stopped flow instrument. RAD-51 D238A alone (1000 nM) or in the presence of indicated amount of RFS-1/RIP-1 was mixed with 5'-Cy3-43mer ssDNA (15 nM). Bar graphs of average Δ Cy3 fluorescence for the RAD-51 alone or in the presence of RFS-1/RIP-1 are presented (mean; errors: s.d.) (**a**: n=4-5, **c**: n=4-5, **d**: n=4-6). **b** Normalized Δ Cy3 fluorescence relative to RAD-51 D238A alone at different RFS-1/RIP-1 concentrations from data shown in **a** is compared to wild type RAD-51 shown in Fig. 2b (mean; errors: s.d.). **e-h** effect of RFS-1 L134P/RIP-1 mutant complex on wild type RAD-51 assembly in the absence of any nucleotide (**e-f**) or in the presence of ATP (**g**) or ADP (**h**). **e, g, h** Average normalized 5'-Cy3-43mer fluorescence profiles plotted as a function of time. The arrow indicates the components of the two syringes rapidly mixed at the 0 s time point in a stopped flow instrument. RAD-51 alone (250 nM) or in the presence of RFS-1 L134P/RIP-1 (50 nM) was mixed with 5'-Cy3-43mer ssDNA (15 nM). Bar graphs of average Δ Cy3 fluorescence for the RAD-51 alone or in the presence of RFS-1 L134P/RIP-1 are plotted (mean; errors: s.d.) (e: n=4-5, g: n=6, h: n=5). **f** Normalized Δ Cy3 fluorescence relative to RAD-51 alone at different RFS-1 L134P/RIP-1 concentrations is compared to wild type RFS-1/RIP-1 shown in Fig. 2b (mean; errors: s.d.). **i** RFS-1/RIP-1 directly interacts with RAD-51. RAD-51 alone (8 μg) or with RFS-1/RIP-1 protein (4 μg) was mixed with anti-FLAG agarose beads in the absence of nucleotide or presence of ATP or ADP. Beads were incubated 30 minutes on room temperature in SF buffer containing 0.1% Tween 20, washed with the same buffer containing 200 mM NaCl and proteins eluted by Laemmli buffer. Representative gel is shown (n=3). Lane 1 represents protein molecular weight marker, lanes 2 and 15 input fractions, remaining lanes are elutions from beads. **j** The amount of RAD-51 pulled down by RFS-1/RIP-1 complex was analysed. The signal of RAD-51 from negative controls missing RFS-1/RIP-1 (lanes 3,4,5 and 9,10,11 from panel **i**) was subtracted from corresponding samples (lanes 6,7,8 and 12,13,14). The RAD-51 enrichment is shown as a ratio of RAD-51 subtracted signal to RIP-1 or RFS-1 signal (mean; errors: s.d., n=3). p-values were obtained by Student's t test (two-tailed): *$p < 0.05$. Source data are provided as a Source Data file.

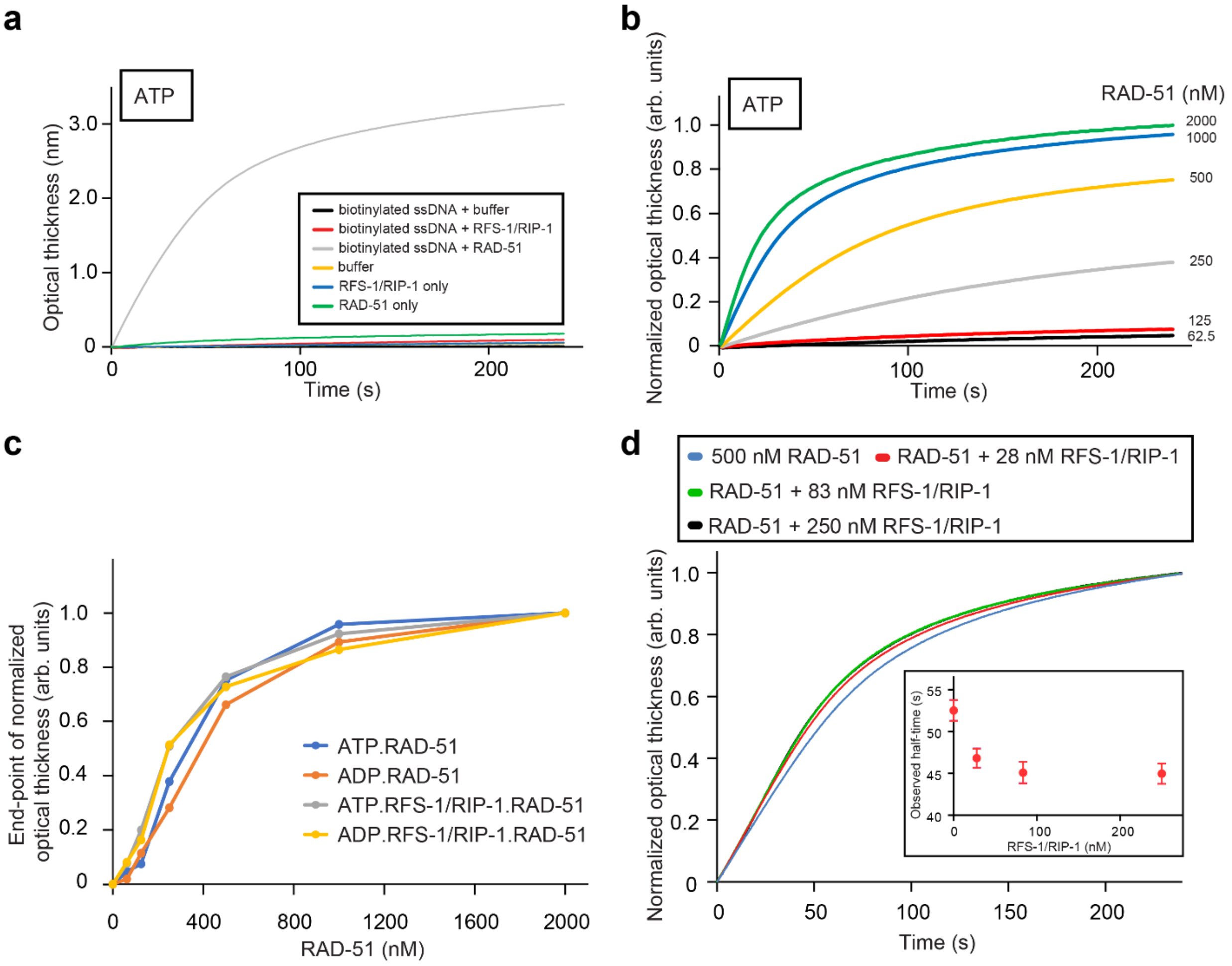

Supplementary Fig. S4 | RAD-51 binds to ssDNA in concentration dependant manner and is modulated by RFS-1/RIP-1. Bio-layer interferometry sensorgrams showing association of proteins on biosensors. **a**, A solution of either RAD-51 or RFS-1/RIP-1 (1000 nM) or buffer only, were loaded on the biosensor coated by 3'-biotinylated ssDNA or without ssDNA. Raw optical thickness values are plotted as a function of time. **b**, A solution of increasing amounts of RAD-51 protein (62.5-2000 nM) was loaded in the presence of ATP onto the biosensor coated with 3'-biotinylated ssDNA. Normalized optical thickness was plotted as a function of time to allow comparison of different conditions. **c**, End-point values of normalized optical thickness for increasing concentrations of RAD-51 with or without RFS-1/RIP-1 (constant molar ratio 4:1) in the presence of ATP or ADP. **d**, RAD-51 (1 μM) was premixed with increasing concentrations of RFS-1/RIP-1 (28-250 nM) and loaded on 3'-biotinylated ssDNA in the presence of ATP. Normalized optical thickness was plotted as a function of time and observed half-times of association are shown on inset chart as a function of RFS-1/RIP-1 concentration (mean; errors: s.d.; n=3). Source data are provided as a Source Data file.

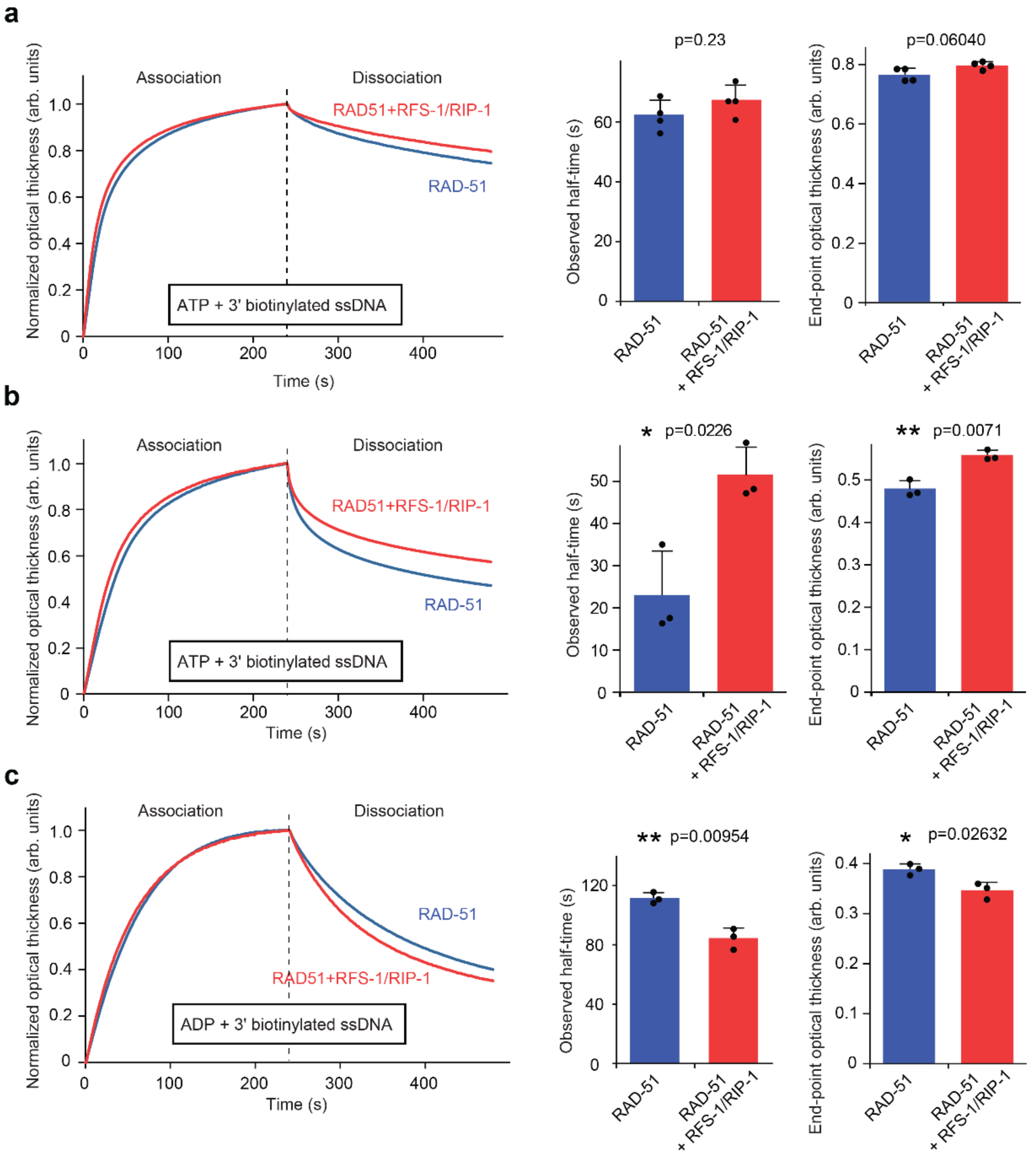

Supplementary Fig. S5 | RFS-1/RIP-1 activities on RAD-51-ssDNA filament are detectable by bio-layer interferometry method. Bio-layer interferometry sensorgrams obtained using 3'-biotinylated ssDNA conjugated to streptavidin-loaded biosensors. **a**, A solution of RAD-51 (1 μM) alone or in the presence of RFS-1/RIP-1 (0.25 μM) was loaded onto the biosensor in the presence of ATP (association phase). At the point indicated by the dashed black line, the biosensor was submerged in buffer with 1000-fold excess of unlabelled ssDNA to promote disassembly of RAD-51 from the immobilized DNA (dissociation phase). **b,** A solution of RAD-51 (1 μM) alone or together with RFS-1/RIP-1 (0.25 μM) was loaded on biosensor in the presence of ATP (association phase). The biosensor was then submerged in buffer with 300 mM NaCl to promote disassembly of RAD-51 from the immobilized DNA (dissociation phase). **c,** A solution of RAD-51 (1 μM) alone or together with RFS-1/RIP-1 (0.25 μM) was loaded on biosensor in the presence of ADP (association phase) at 50 mM NaCl. The biosensor was then submerged in buffer to promote disassembly of RAD-51 from the immobilized DNA (dissociation phase). Observed half-times and end-point optical thickness for dissociation phases are presented in corresponding bar graphs (mean; errors: s.d.; a: n=4, b: n=3, c: n=3). p-values were obtained by Student's t test (two-tailed): $*p < 0.05$; $**p < 0.01$. Source data are provided as a Source Data file.

Supplementary Table 1.
Oligonucleotides used in the study.

5'-Cy3-43mer	5'-Cy3-TTTTTTTTTTTTTTTTTTTTTTTTTTTTTTTTTTTTTTTTTTT
3'-Cy3-43mer	TTTTTTTTTTTTTTTTTTTTTTTTTTTTTTTTTTTTTTTTTTT-Cy3-3'
(Int21)-Cy3-43mer	TTTTTTTTTTTTTTTTTTTTT-Cy3-TTTTTTTTTTTTTTTTTTTTTT
Unlabeled 43mer	TTTTTTTTTTTTTTTTTTTTTTTTTTTTTTTTTTTTTTTTTTT
5'-FITC-61mer	5'-FITC-GACGCTGCCGAATTCTACCAGTGCCTTGCTAGGACATCTTT GCCCACCTGCAGGTTCACCC
Unlabeled 61mer	GACGCTGCCGAATTCTACCAGTGCCTTGCTAGGACATCTTT GCCCACCTGCAGGTTCACCC
3'-biotinylated ssDNA	5'-Biotin-TTTTTTTTTTTTTTTTTTTTTTTTTTTTTTTTTTTTTTTTTTT
