# Supplementary material for: Nucleotide proofreading functions by nematode RAD51 paralogs facilitate optimal RAD51 filament function
Source: Nat Commun. 2021 Sep 20;12:5545. doi: 10.1038/s41467-021-25830-x (PMC8452638; doi:10.1038/s41467-021-25830-x)
Supplement: Supplementary file 3 — Reporting Summary [file 41467_2021_25830_MOESM3_ESM.pdf]

nature portfolio

Corresponding author(s): Lumir Krejci

Last updated by author(s): Sep 2, 2021

Reporting Summary

Nature Portfolio wishes to improve the reproducibility of the work that we publish. This form provides structure for consistency and transparency in reporting. For further information on Nature Portfolio policies, see our Editorial Policies and the Editorial Policy Checklist.

Statistics

For all statistical analyses, confirm that the following items are present in the figure legend, table legend, main text, or Methods section.

n/a	Confirmed	
☐	☒	The exact sample size (n) for each experimental group/condition, given as a discrete number and unit of measurement
☐	☒	A statement on whether measurements were taken from distinct samples or whether the same sample was measured repeatedly
☐	☒	The statistical test(s) used AND whether they are one- or two-sided<br>*Only common tests should be described solely by name; describe more complex techniques in the Methods section.*
☒	☐	A description of all covariates tested
☒	☐	A description of any assumptions or corrections, such as tests of normality and adjustment for multiple comparisons
☐	☒	A full description of the statistical parameters including central tendency (e.g. means) or other basic estimates (e.g. regression coefficient) AND variation (e.g. standard deviation) or associated estimates of uncertainty (e.g. confidence intervals)
☐	☒	For null hypothesis testing, the test statistic (e.g. F, t, r) with confidence intervals, effect sizes, degrees of freedom and P value noted<br>*Give P values as exact values whenever suitable.*
☒	☐	For Bayesian analysis, information on the choice of priors and Markov chain Monte Carlo settings
☒	☐	For hierarchical and complex designs, identification of the appropriate level for tests and full reporting of outcomes
☒	☐	Estimates of effect sizes (e.g. Cohen's d, Pearson's r), indicating how they were calculated

Our web collection on statistics for biologists contains articles on many of the points above.

Software and code

Policy information about availability of computer code

Data collection	BioLogic Bio-kine32 V4.80<br>Carestream Molecular Imaging Software SE v5.3.2.16673<br>ForteBio BLItz Pro 1.2.1.5<br>ForteBio Data Acquisition v11.1.1.19
Data analysis	Fujifilm MultiGauge V3.2<br>Microsoft Excel 365

For manuscripts utilizing custom algorithms or software that are central to the research but not yet described in published literature, software must be made available to editors and reviewers. We strongly encourage code deposition in a community repository (e.g. GitHub). See the Nature Portfolio guidelines for submitting code & software for further information.

Data

Policy information about availability of data

All manuscripts must include a data availability statement. This statement should provide the following information, where applicable:

- Accession codes, unique identifiers, or web links for publicly available datasets
- A description of any restrictions on data availability
- For clinical datasets or third party data, please ensure that the statement adheres to our policy

The data supporting the findings of this study are available within the article, its Supplementary Information files, and the Source Data file provided with this paper.

Field-specific reporting

Please select the one below that is the best fit for your research. If you are not sure, read the appropriate sections before making your selection.

☒ Life sciences ☐ Behavioural & social sciences ☐ Ecological, evolutionary & environmental sciences

For a reference copy of the document with all sections, see nature.com/documents/nr-reporting-summary-flat.pdf

Life sciences study design

All studies must disclose on these points even when the disclosure is negative.

Sample size	Sample sizes were not predetermined based on statistical methods, but were chosen based on the our previous studies in which similar experiments were conducted (Taylor et al, Cell, 2015; Taylor et al, Mol Cell, 2016). The numbers of independent experiments are indicated in the figure legends.
Data exclusions	No data were excluded from analyses in the experiments, except in the case we observed technical artefacts caused by stop-flow machine malfunction.
Replication	All experimental results were reliably reproduced. At least three independent replications for assays were conducted, and noted in the figure legend. Major findings were reproduced with new batch of purified proteins.
Randomization	We have conducted the experiments with different batches of oligonucleotides and proteins, major observations were repeated on two different types of stop-flow machine and by three different people (Spirek, Taylor and Belan) - experiments always had reproducible results.
Blinding	Blinding is not required as all data was collected by objective measurements.

Reporting for specific materials, systems and methods

We require information from authors about some types of materials, experimental systems and methods used in many studies. Here, indicate whether each material, system or method listed is relevant to your study. If you are not sure if a list item applies to your research, read the appropriate section before selecting a response.

Materials & experimental systems

n/a	Involved in the study	
☒	☐	Antibodies
☒	☐	Eukaryotic cell lines
☒	☐	Palaeontology and archaeology
☒	☐	Animals and other organisms
☒	☐	Human research participants
☒	☐	Clinical data
☒	☐	Dual use research of concern

Methods

n/a	Involved in the study	
☒	☐	ChIP-seq
☒	☐	Flow cytometry
☒	☐	MRI-based neuroimaging
